# P-173. Investigation of a food poisoning outbreak at a holiday event in Ekibastuz, Kazakhstan, 2024

**DOI:** 10.1093/ofid/ofaf695.397

**Published:** 2026-01-11

**Authors:** Dilnaz Aitbaeva, Aidana Tulemagambetova, Ulyana Gubareva, Saya Gazezova, Ainagul Kuatbaeva, Roberta Horth, Dilyara Nabirova

**Affiliations:** Central Asia FETP, Almaty, Almaty, Kazakhstan; Central Asia FETP, Almaty, Almaty, Kazakhstan; Central Asia FETP, Almaty, Almaty, Kazakhstan; Central Asia Field Epidemiology Training Program, Almaty, Almaty, Kazakhstan; Scientific and Practical Center of Sanitary-Epidemiological Expertise and Monitoring Almaty, Kazakhstan, Almaty, Almaty, Kazakhstan; US Centers for Disease Control and Prevention, Dulles, Virginia; CDC Central Asia office, Almaty, Almaty, Kazakhstan

## Abstract

**Background:**

From April 14–20, 2024, in Ekibastuz, Kazakhstan, 94 people who ate at the same restaurant on April 13 sought medical care for acute gastroenteritis. We aimed to confirm the outbreak and identify the source.

**Table 1.**
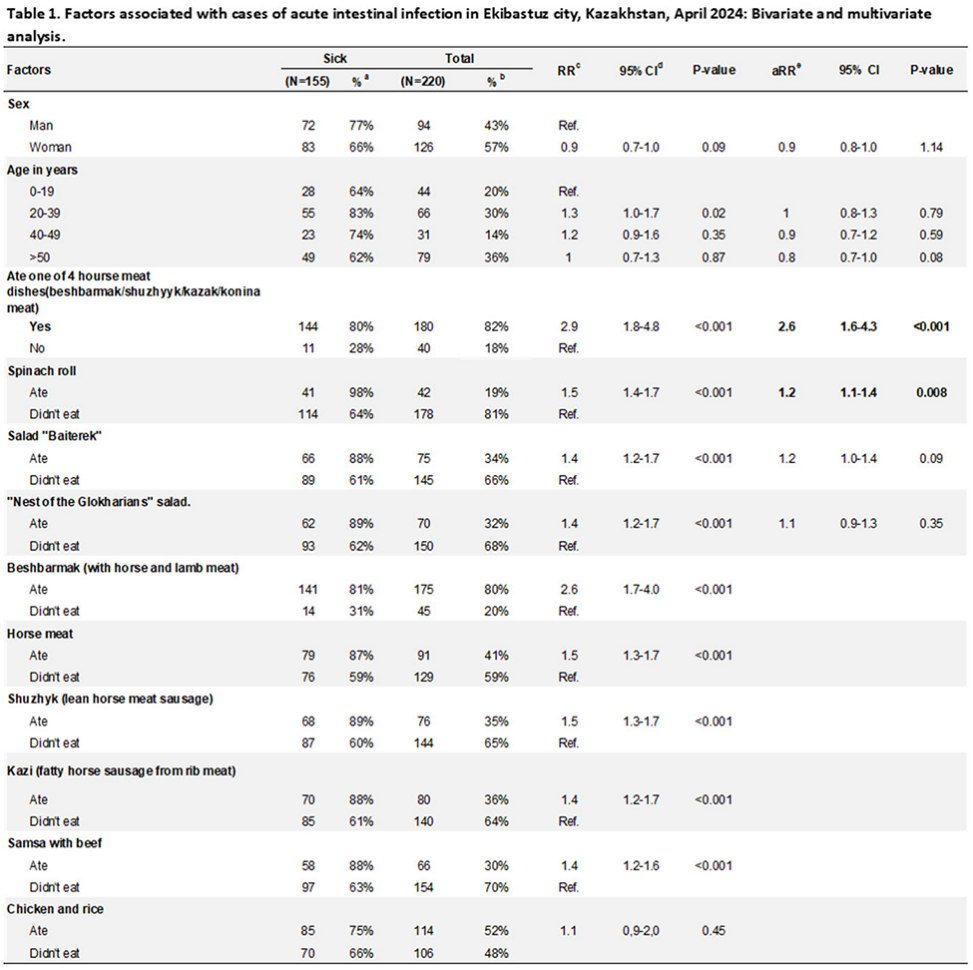
Factors associated with cases of acute intestinal infection in Ekibastuz city, Kazakhstan, April 2024: Bivariate and multivariate analysis. a: row percent b: column percent c: RR = Risk ratio, d: CI = Confidence Interval e: aRR = adjusted Risk ratio

**Methods:**

The restaurant provided a list of 257 people who attended two events on April 13. Using a retrospective cohort design, we interviewed consenting attendees. Participants were classified as ill if they had symptoms or sought healthcare for food poisoning (diarrhea, fever ≥37.5°C, vomiting). Restaurant surfaces, leftover foods, and stool samples (from hospitalized patients and restaurant staff) were tested. We performed Poisson regression to calculate adjusted risk ratios of illness (RR) and 95% confidence intervals (CI).

**Results:**

Among 220 attendees, 155 were ill, with 92% experiencing diarrhea, 80% fever, 66% vomiting, and 29% hospitalized. Symptom onset ranged from 3–24 hours after eating at the restaurant. Most ill participants were 20–39 years old (83%) and male (77%). Risk was elevated among attendees eating potato/rice side dishes (55% of ill and 48% of non-ill attendees; RR=1.4; 95% CI=1.2–1.7). Horsemeat-containing dishes (consumed by 93% of ill and 55% of non-ill attendees) had increased risk compared with no horsemeat (RR=2.9; 95% CI=1.8-4.8). Increased risk was associated with eating beshbarmak (RR=2.6; 95% CI=1.7–4.0), shuzhuk (RR=1.5; 95%CI=1.3–1.7), and qazi (RR=1.4; 95% CI=1.2–1.7). In multivariable analysis, the risk of illness from eating any horsemeat-containing dishes versus no horsemeat was 2.6 (95%CI=1.6–4.3). In hospitalized participants (n=45) and restaurant staff (n=4), Salmonella enteritidis was detected in 98% and Escherichia coli in 60%. S. enteritidis and E. coli were found in potato/rice side-dishes and E. coli in horsemeat leftovers and food preparation surfaces.

**Conclusion:**

Multiple pathogens might have contributed to the outbreak through multiple sources within the restaurant. This underlines the need for restaurant owners to ensure strict compliance with hygiene standards and implement education programs on hygienic and proper cooking practices to prevent future outbreaks.

**Disclosures:**

All Authors: No reported disclosures

